# Scanning, Contextual Factors, and Association With Performance in English Premier League Footballers: An Investigation Across a Season

**DOI:** 10.3389/fpsyg.2020.553813

**Published:** 2020-10-06

**Authors:** Geir Jordet, Karl Marius Aksum, Daniel N. Pedersen, Anup Walvekar, Arjav Trivedi, Alan McCall, Andreas Ivarsson, David Priestley

**Affiliations:** ^1^Department of Sport and Social Sciences, Norwegian School of Sport Sciences, Oslo, Norway; ^2^Arsenal Psychology and Research Group, Arsenal Football Club, London, United Kingdom; ^3^National University of Singapore, Singapore, Singapore; ^4^Arsenal Performance and Research Team, Arsenal Football Club, London, United Kingdom; ^5^School of Applied Sciences, Edinburgh Napier University, Edinburgh, United Kingdom; ^6^Halmstad University, Halmstad, Sweden

**Keywords:** soccer (football), perception, decision making, vision, visual search, exploration

## Abstract

Scanning in football (soccer) denotes an active head movement where a player’s face is temporarily directed away from the ball to gather information in preparation for subsequently engaging with the ball. The aim of this study was to learn more about the ways that 27 elite professional football players in an English Premier League club use scanning in competitive matches, the conditions under which this behavior is exhibited, and the relationships between these behaviors and performance. Players were filmed across 21 matches, producing a total number of 9,574 individual ball possessions for analysis. Close-up video analyses of scanning show positional differences (with central midfielders and central defenders scanning most frequently, forwards least) and contextual differences (with relatively lower scanning frequency in situations with tight opponent pressure, in positions wide in the field and closer to the opponent’s goal, and under certain game state conditions). Players scan more frequently prior to giving passes than when they dribble, shoot, or only receive it, as well as prior to more long/forward passes compared to short/backward ones, although these differences are small. A Bayesian hierarchical model, which accounts for individual player differences and pass difficulty, suggests that the more a player scans, the higher the probability of completing a pass. In conclusion, match demands are likely to constrain the extent to which highly elite players scan, and scanning seems to have a small, but positive role in elite football players’ performance.

## Introduction

Football (soccer) is a highly dynamic, fluid, and complex sport, and players’ ability to pick up and use visual information from teammates and opponents may, logically, be a key to performance. Indeed, researchers have uncovered perceptual and cognitive mechanisms that differentiate skilled from less skilled football players, and superior from inferior performances (for recent reviews, see Mann et al., 2019; Williams and Jackson, 2019). Much of this research has, however, examined visual search strategies. Typically, these studies are carried out with eye tracking devices where players view and respond to photographs or video films positioned in front of them in a laboratory setting. Some of these studies reveal that skilled football players fixate their gaze less frequently, but with longer durations, which may imply that they are able to extract more information from each individual visual fixation (Helsen and Starkes, 1999; Cañal-Bruland et al., 2011). Other studies show that skilled football players fixate their gaze on the displayed information more frequently, but with shorter duration (Vaeyens et al., 2007a,b; Roca et al., 2011). Recently, for example, in a study of 44 professional and semiprofessional players in England (Roca et al., 2018), the most creative players adopted a broader attention span, by showing more visual fixations of shorter duration than the less creative players. This frequent change in fixation location makes sense as, in team ball sports, players are required to shift attention between different objects, most notably between the ball and other players (Jordet, 2005a,b; Mann et al., 2019). With that said, all these studies have been conducted in laboratories, and it is possible that variations in the extent to which the experimental setup resembles the real world (i.e., the degree of representative design, see Araújo et al., 2007; Pinder et al., 2011) could account for the different results. Indeed, a substantial gap in the literature on perceptual and cognitive processes in sport is the lack of research focusing on what athletes are doing on the field in real competitive events (outside the laboratory).

In addition, very few studies have documented perceptual processes of truly elite, professional players, possibly because this population is difficult to recruit for this type of research. Thus, another line of research has started from the other direction than the laboratory visual search studies, by systematically observing and analyzing elite football players’ behaviors in actual, real-world games. This relatively new paradigm is based upon the ecological theories by Gibson (1966, 1979), who argued that perception is an active process of obtaining information from the world, a psychosomatic act, consisting of motor action. Exploratory activity is activity initiated to detect information (Gibson, 1966, 1979). More specifically, exploratory activity denotes “the scanning for and use of information [that] involves adjustment of the head and sensory organs to the ambient energy fields” (Reed, 1996, p. 80). In football, this activity is sometimes referred to using other terms. For example, in German football, they use the word “vororientierung” (e.g., Scheibe, 2019), which translated to English would be “pre-orientation” (specifying that this activity takes place prior to receiving the ball). In English, coaches often refer to this activity as “checking your shoulder” or “scanning.” With respect to empirical research on this activity, Jordet (2005a) was first to film professional football players with high-zoom video cameras to obtain close-up images of each individual player, making it possible to examine details in the players’ scanning behavior leading up to receiving the ball. It was shown that for midfielders, engaging in successive scanning of the areas of the field behind one’s back seemed a necessary foundation to subsequently perceive and successfully act upon information located in these areas. In the most extensive research report to date, Jordet et al. (2013) obtained and analyzed Sky Sport’s PlayerCam broadcasts of 1,279 game situations with 118 football players (midfielders and forwards) in the English Premier League (EPL). The players in this sample who at some point had received a prestigious individual award (e.g., FIFA World Player of the Year) scanned more frequently than others prior to receiving the ball, and there was a positive relationship between scanning frequency and pass completion. Similarly, in a study of three youth elite, midfield players, it was found that when the players showed any scanning behaviors prior to receiving the ball (compared to the ones who did not show any such behavior), they performed more forward passes, executed more passes into the attacking half, performed more turns when opportunities arose, and experienced less defensive pressure from opponents (Eldridge et al., 2013). However, there was no significant relationship found between scanning and maintained possession of the ball.

Additionally, attempts have been made to analyze football players’ head movements using wearable inertial measurement units, worn in a headband at the back of players’ heads. Results show that higher scanning frequency before possession (i.e., measured as all registered head turns, thus not necessarily linked to directing one’s face toward areas located away from the ball) is associated with faster passing response time (McGuckian et al., 2019) and higher likelihood of forward passes (McGuckian et al., 2018). However, there was no relationship with pass success in any of these studies. Further, higher head turn excursion (i.e., degrees of head turning) was associated with higher likelihood of turning with ball and switching play, whereas lower excursion was associated with higher likelihood of performing one-touch passes (McGuckian et al., 2018). Finally, it has been found that youth elite players scanned more extensively when in possession of the ball than without the ball, more in the back third of the pitch and least in the middle third of the pitch, and players in more central roles scanned more extensively than players in wider roles when they themselves, or their team, had possession of the ball (McGuckian et al., 2020).

However, none of these field-based studies have sufficiently controlled statistically for contextual and personal factors that may influence these results, and it seems paramount to examine the impact of such factors. One example of considerable contextual influence on scanning could be interpreted from a study comparing futsal and football players, where the scene camera of a mobile eye tracker was used to collect data on attention orientation during a 5-v-5 small-sided game setup (Oppici et al., 2017). It was found that the futsal players focused their attention toward other players during ball reception and control, whereas the football players scanned more toward other players when they were not involved with the ball (and their team was in possession of the ball).

To summarize, there is evidence for some contextual variation with respect to football players’ visual scanning, and there seem to be some performance benefits of engaging in scanning prior to receiving the ball. However, there is still very limited knowledge about how elite, professional football players employ scanning behaviors in actual real-world games. Additionally, researchers have typically relied on a relatively small number of observations, using less robust statistical methods that do not account for contextual and personal variation. We expect that the use of more sophisticated statistical analyses better will reveal the relationships between scanning, situational context, and performance. Thus, the aim of this study was to learn about how elite professional football players use visual scanning in real games; establish the extent to which scanning varies under different contextual conditions (e.g., positional role, opponent pressure, pitch location, and game states); and to examine the relationships between scanning and performance. Within this scope, and following discussions with professional coaches at the club this study was carried out at, the main hypothesis that we wanted to test is: Scanning plays a role in successfully completing passes, when we sufficiently control for personal and contextual variation. In addition, following tendencies found in previous studies, we hypothesized that players in central positional roles and locations in the pitch would scan more than players in more peripheral roles and locations, that players under low opponent pressure would scan more than players under high opponent pressure and that scanning would be linked to more forward actions in the field.

## Materials and Methods

### Data and Participants

Participants were 27 professional male football players aged 17–32 years (*M* = 25.66 ± 4.26). All players represented the same team in the EPL in the 2017/2018 season. The data consisted of individual player ball possessions registered in 21 home games (13 Premier League games, 6 UEFA Europa League games, and 2 League cup games) that we filmed that season. This totaled 9,574 ball possessions. The study was reviewed and approved by the Norwegian Centre for Research Data (NSD)—project number 57718. Written informed consent for participation was not required.

### Procedures

The matches were video recorded with three 4K video cameras: two Blackmagic Micro Studio Camera 4K (frequency of up to 59.94 fps) and one Panasonic AG-UX90 4K Camcorder (frequency of up to 60 fps). All cameras were set up and operated by one of the co-authors at a designated camera platform, positioned up in the stands at the mid-point of the touchline. Each Blackmagic camera was fixed to cover one half of the pitch, while the Panasonic camera was manually panned from side to side to cover the ball and as many of the players on the pitch as possible. Following game completion, the video recordings were transferred onto portable hard drives.

We then merged the recordings of each of the two halves from the two Blackmagic cameras together using homography transformations and Opencv package in Python 3.6 and combined this recording with field location coordinates that were hand-tagged by match analysts working at the club using their proprietary software. We used these coordinates to create a Python program that automatically kept the targeted player in the middle of the screen, while zooming in on him to provide a close-up video recording of that player. For the coding of the behaviors on the videos, we created a web-based program using PHP and javascript. When the coders logged on to this program online, they first selected the game and player, and a list of the situations with that player in that game would appear. When they selected a situation, they would automatically see the close-up recording of that player at the left of their screen and an overview video recording of the game (from the Panasonic camera) at the right of the screen. Both these videos were synced at frame level, i.e., the coder could only play the videos together at the same rate. The program recorded keystrokes that were assigned to different variables along with the exact time in the video. In order to obtain the precise time, the user could also move the video forward/backward by one frame at a time. The program also allowed coders to correct their coding by undoing the previous step.

After permission was obtained from the club to film games, we conducted several tests of the filming procedure to first arrive at an effective way to capture such data, and second to ensure the quality of the video recordings. In total, prior to the actual data collection, two initial pilot games were filmed at another stadium and another five test games at the Premier League club stadium. Following this testing, we successfully filmed the remaining home games of the season (except three games, two in the Europa League, and one in the Carabao cup, played during the testing phase, early in the season, which were not prioritized at the time and hence not filmed). One additional game was filmed, but a technological error with one of the cameras precluded the analysis of this particular video recording. Generally, conducting a data collection of this magnitude at a Premier League club is a vast logistical undertaking. Space limitations make it difficult to describe every single aspect of our procedures here, but people interested in replicating our study or our methods are encouraged to contact the lead author who will be able to answer any questions about the procedures.

Manually coding behaviors from distance video recordings of a dynamic and complex real-world event (such as a football game) is unlikely to produce fully objective data, and it was important for us to strive for as much rigor as possible in these analyses. Ultimately, eight students manually coded the behavioral data coming from the videos. These coders comprised of five students in football coaching at the Norwegian School of Sports Sciences and three students from different American universities who all had in common that they attended the 2018 MIT Sloan Sport Analytics conference in Boston, MA. Everyone was trained in the procedures, where they coded a selection of situations and received feedback by an experienced coder. Only when their coding would yield a total agreement of at least 80% with one of the experienced coders on all tested variables (80% cutoff for coding of behavioral data has previously been used as an acceptable threshold in sport psychology, Hrycaiko and Martin, 1996) was the person allowed to code the data that would be used for further analysis. After the actual analyses had commenced, we continued to test the interrater reliability for all coders and on all behavioral variables. An experienced coder (who had completed a master’s thesis on the topic of visual perception in football, had background as a professional football player and was also used to train the student coders) blindly coded a total of 784 randomly selected ball possessions previously coded by the eight coders. To assess the interrater reliability, we followed the recommendations from Hallgren (2012) and calculated Cohen κ for nominal variables and intraclass correlations (ICCs) for ordinal, interval, and ratio variables. For the primary variable in our study, scanning, we found the following ICC coefficients between each of the eight coders and the expert coder (in descending order): 0.993, 0.991, 0.988, 0.986, 0.982, 0.981, 0.937, and 0.825 [mean (*M*) = 0.960, standard deviation (SD) = 0.058]. Based on suggested cutoffs all these scores were considered excellent (Cicchetti, 1994). The coder who had considerably lower ICC values than the others (at 0.825) was followed up throughout with extra feedback and training. Although his ICC score could still be considered more than acceptable, we decided to stop his work (yet retain his coding for the analyses). Of all the coders, he was the one who coded the fewest ball possessions, with a total of 200 possessions coded. Also, in an early phase of the coding process, there was one more individual who passed the training phase and started coding, but whose personal ICC values were even lower, at 0.515 in total. Even though this value is considered “fair” (following Cicchetti, 1994), we decided to stop the work with this coder, cut all the possessions that had been coded by this individual (329 possessions in total), and have another coder recode those possessions. For the aggregated reliability results, see section *Interrater Reliability*.

### Variables

There were three categories of variables in this study, those related to scanning, context, and performance with the ball.

#### Scanning

In ecological psychology literature (e.g., Gibson, 1979), “exploration,” “exploratory behavior,” or “exploratory activity” are the preferred terms, while in more cognitively oriented literature (e.g., Mann et al., 2019) “visual search” is more used. In this article, unless we are referring to specific theoretical or empirical work where sticking to their original term is of importance, we will refer to this activity as “scanning.”

##### Scan

A scan was operationally defined as a player’s active head movement where the face (and hence, the eyes) is temporarily directed away from the ball, with the assumed intention of gathering information about teammates and/or opponents, to prepare for subsequently engaging with the ball (based on Jordet, 2005b).

##### Scan frequency

Scan frequency is the number of scans per second, measured in the last 10 s that the team possessed the ball, before the target player received the ball. The 10-s cutoff has been used in previous studies on scanning in football players (e.g., Jordet et al., 2013; McGuckian et al., 2018). If within that 10-s time interval, the other team had possession and lost it to the target player’s team, the time interval would instead start at the moment possession was won and end with the target player receiving the ball. Ball possession was here defined as having control of the ball. In instances where the opponent team was in contact with the ball one or two times without having control (typically when clearing the ball out, dueling for the ball, or deflecting a pass), the target player’s team had not lost possession in our analyses. For set plays (e.g., a free kick or a throw-in) within the 10-s interval, the time interval for measuring scans was set from 2 s before the ball was put in play (to allow some time to register scanning prior to the ball is in actual play), and end when the target player would receive the ball.

#### Context

##### Positional role

The positional roles were categorized into central defender, side defender, central midfielder, winger, and forward. This categorization was based on the official line-up for each game (disclosed by the club) indicating the positions held by the players at the start of the game. This was then verified with the exact average *x*, *y* position on the pitch that each of the players was located at in each game (also publicly disclosed by the club, on their website). Thus, if a player changed position during the match, his involvement would still be coded in the playing position he had for the beginning and/or most of the match.

##### Pitch location

Pitch location is defined as the player’s position on the pitch when receiving the ball from a pass. Pass distance is calculated as the difference between the location of a pass and its reception. The *x*, *y* coordinates of the pass event (and reception) were hand-tagged by the club’s professionally trained coders (StatDNA LLC). Trained coders simultaneously view broadcast footage with a pitch map, and they tag onto the pitch the approximate *x*, *y* coordinates of a pass event and its reception using proprietary tagging software.

Optical tracking data (e.g., TRACAB^®^; Linke et al., 2020) could have provided a higher resolution alternative, but it was not available in our dataset. It is important to note that there is no ultimate “ground-truth” for positional data, because as yet there is no tracker inside the football (or universally worn by all players) to accurately measure their pitch position in real-game situations. As a result, there will always be some degree of measurement error, and here we relied upon a twofold quality assurance (QA) process in our data collection to attempt to mitigate this. First, automated tagging software detects and flags any unrealistic positional values (e.g., passes made that originate out-of-bounds and are not set pieces). This is followed by a QA evaluator rechecking the coded data to ensure reasonable values.

##### Opponent pressure

Opponent pressure was operationally defined as the distance between the target player and the closest opponent, at the moment the target player received the ball (measured in meters). This was visually assessed for each ball possession by the student coders. The coders were trained in using a variety of reference points to facilitate reliable assessments of these distances, such as the length and width of the pitch, the distances between different lines and markings on the pitch, and the width and length of the checkered/striped pattern in the grass on the pitch (all in exact meters).

##### Game state

We assessed game state in two basic ways: game standing and accumulated game time. Game standing denotes whether the team, at the moment of that particular ball possession, is winning (i.e., ahead in the stand, such as 1–0, 2–1 or 2–0), losing (i.e., behind in the stand, such as 0–1, 0–2 or 1–2), or drawing (i.e., the stand is tied, such as 0–0, 1–1 or 2–2). Accumulated game time was assessed using 5-min time intervals (from 0 to 90 min, including a category for added time to each half, so 45+ and 90+ min). To capture real accumulated game time for each player, only the players who started the game were included in this particular part of the analysis.

#### Performance With the Ball

##### Action direction

This variable assesses the direction of the target player’s action in each situation, where the direction is estimated by the final position of the ball after the end action (as a player may move in several directions while being in possession of the ball) in relation to the opponent’s goal line. Forward action is when the ball (e.g., from a pass or dribble) ends up closer to the opponent’s goal line; backward action is when the ball ends up further from the opponent’s goal line; sideward action is when the ball ends up approximately at the same distance from the opponent’s goal line. Only vertical direction was measured in this variable, and possessions were only categorized as sideward in those instances where we could not say for sure that it was either forward or backward. The coders were trained in using the checkered/striped pattern in the grass on the pitch as a reference when assessing whether an action was forward/backward or sideward.

##### Action type

The types of last actions registered were pass, shot, dribble, and receiving (where the latter typically, but not always, would imply that the ball was lost in the act of receiving or attempting to receive). The types of passes registered were long penetrative pass (passing two or more lines of the opposition defense, where a line could be the forward line, midfield line, and defensive line), short penetrative pass (passing one line of defense), forward non-penetrative pass (forward in the field, but not passing any defensive line), sideward pass (neither forward nor backward), backward pass, and no-pass (where the last action registered was a shot, dribble, or receiving the ball).

##### Successful actions

If the team of the target player maintains possession after the player’s last action with the ball, this is registered as a successful action (although we do not claim that this would be the right action in view of a coach). Typically, this is a pass that reaches a teammate (i.e., pass completion), but it could also be a shot that is scored or a dribble or receiving action that produces continued possession (via a deflection so the ball goes to a teammate or a won throw-in). If the ball goes to an opponent (e.g., a pass that is intercepted, a failed dribble, a shot that goes wide of the goal, or a failed attempt to receive the ball), thus possession is not maintained, it is registered as an unsuccessful action.

### Statistical Analysis

#### Interrater Reliability

For number of scans (the basis of the variable Scan frequency), we did double coding to assess interrater reliability on 784 of the total 9,574 individual ball possessions (8.2%). The resulting overall ICC was 0.979 (*p* < 0.001), which is considered “excellent” agreement (Cicchetti, 1994). For the other variables, 166 (1.7%) of these possessions were analyzed double. For Opponent pressure, which also is a continuous variable, the ICC coefficient was 0.981 (*p* < 0.001) (indicating “excellent” agreement). For the remaining variables that were all categorical, we estimated κ values, and all agreements were considered “almost perfect”: pass type *k* = 0.867 (*p* < 0.001), action type *k* = 0.851 (*p* < 0.001), action direction *k* = 0.916 (*p* < 0.001), and successful action *k* = 0.978 (*p* < 0.001) (Cohen, 1960; Landis and Koch, 1977).

#### Descriptive Analyses

The initial part of the statistical analyses was performed using SPSS (version 24). First, to test whether the scanning variable was normally distributed a Kolmogorov-Smirnov test was performed. Because the result showed that average scanning frequency significantly deviated from normal distribution (*D* = 0.07, *p* < 0.001), non-parametric tests were used. Second, the Kruskal–Wallis test in combination with the Dunn multiple comparison *post hoc* test were used to analyze differences in scanning behaviors under different contextual conditions (e.g., positional role, opponent pressure, pitch location, and game states). Bonferroni adjustments were conducted to control for the multiple testing procedure. Third, the Mann-Whitney *U* test was used to analyze differences in scan frequency between successful and unsuccessful actions. Fourth, for all analyses, Cohen *d* effect sizes were calculated to indicate the magnitude of the effects for each of the pair-wise comparisons, where we will discuss values that are above 0.20 (considered a small effect), above 0.50 (medium effect), and above 0.80 (large effect) (based on Cohen, 1988).

### Modeling Pass Completion Using Scanning as a Predictor Variable

#### Hierarchical Bayesian Model With a Single Explanatory Variable

##### Motivation

We want to model the outcome of a pass using scanning as a predictor variable, to quantify whether it has a credible non-zero effect. To motivate our model selection, we first note that our observations of passes are *not* independent of one another, because different players pass the ball multiple times.

Player identity may play a role in pass completion in two ways. First, players have varying technical abilities: some are better at completing passes than others. Second, players may have different scanning tendencies, we may or may not find that when a player scans more (relative to their baseline), they may also have a higher probability of pass completion. The rate of improvement may be the same or varying across all players. Any pass completion model using scanning as a variable ought to account for individualized player effects.

As such, we chose to fit a hierarchical Bayesian model [see Model Description (both sections under Hierarchical Bayesian Model With a Single Explanatory Variable and Hierarchical Bayesian Model With Multiple Explanatory Variables], using the “pymc3” Python package (Salvatier et al., 2016), to estimate individualized player scanning coefficients. These are modeled as parameters sampled from an overall (“group”) scanning distribution.

This approach has the added benefit of accounting for varying observational sample sizes between players. When estimating individualized player scanning coefficients, we split observations by player. However, some players have fewer scanning observations. A hierarchical Bayesian approach accounts for this through *shrinkage*: when there are fewer observations, the individualized player distribution tends to the overall group distribution.

Additionally, the Bayesian interval estimator is given by a “credible” interval (rather than a “confidence” interval), directly understood as a probabilistic measure of uncertainty around the true value of the coefficient.

##### Model description

**FIGURE 1:**
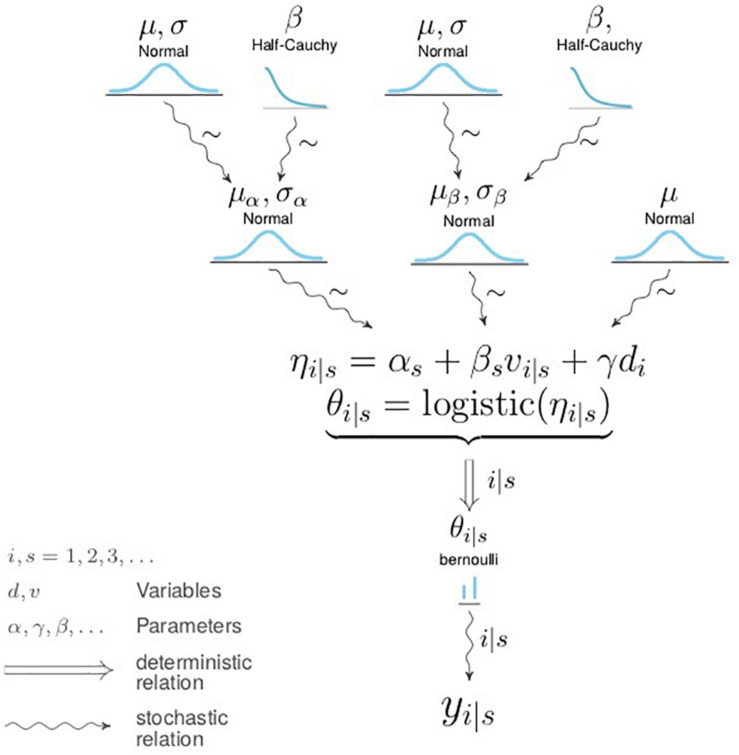
Diagram showing the full Bayesian hierarchical model.

The pass outcome, *y*_*i*_, of the *i*th pass, is observed as complete (*y=1*) or incomplete (*y=0*), and *v*_*i*_ is the search frequency before the *i*th pass. We assume each pass is a Bernoulli trial, where *y*_*i*_ = 1 with probability *p*_*i*_ and *y*_*i*_ = 0 with probability 1-*p*_*i*_. We modeled the outcome *y*_*i*_ using a hierarchical logistic regression (c.f. Figure 1; without the γ term) as follows:

(1) $$\eta_{i|s}=\alpha_{s}+\beta_{s}\,v_{i|s}$$

where η_*i—s*_ is the log-odds of pass completion for the *i*th pass by player *s* (the “subject”), and *v*_*i—s*_ is the scanning frequency of that pass. α_*s*_ is the intercept term, varying for every player and accounting for their baseline technical ability. β_*s*_ is the scanning coefficient, varying for every player. There are 27 players in our dataset, therefore we will be estimating 27 α_*s*_ and 27 β_*s*_ terms, one pair per player. For this and the next model (see section *Hierarchical Bayesian Model With Multiple Explanatory Variables*), the independent variables were standardized by their mean and SD.

As we are interested in the overall group-level effect of scanning, we assume that the α_*s*_ and β_*s*_ coefficients are themselves normally distributed as follows:

(2) $$\begin{array}{c} \alpha_{s}∼\mathrm{Normal}\,\left(\mu_{\alpha },\sigma_{\alpha }\right)\\ \beta_{s}∼\mathrm{Normal}\,\left(\mu_{\beta },\sigma_{\beta }\right) \end{array}$$

where μ_α_,σ_α_ and μ_β_,σ_β_are group-level parameters describing the overall distribution of individual technical ability and scanning tendencies respectively. We set the prior distributions on μ_α_,μ_β_ as follows:

$$\mu_{\alpha },\mu_{\beta }∼N\,o\,r\,m\,a\,l\,\left(0,1\right)$$

We chose these priors as we have no reason to believe that they are not continuous variables defined over the infinite range [−∞, + ∞]; furthermore, many natural phenomena are modeled with Normal distributions, so we find it reasonable to assume that the effect of scanning (and baseline technical ability) is also normally distributed. The strength of these priors is weakly informative, but suitably so as to not unduly influence the posterior parameter distributions: given that when the log odds η≈2.2, *p*≈0.9, and when η≈−2.2, *p*≈0.1, our scale parameter = 1 and does not constrain us tightly around our location parameter (= 0).

As we are unsure of the magnitude of the variance parameter, we set vague uninformative priors on σ_α_,σ_β_ as follows:

$$\sigma_{\alpha },\sigma_{\beta }∼Half-Cauchy\left(\beta =25\right)$$

in accordance with Gelman (2006).

For this model and the next (see section *Hierarchical Bayesian Model With Multiple Explanatory Variables*), the pymc3 NUTS sampler (“No U-Turn Sampler”) was used to generate samples. Unless otherwise stated, for each model, four chains (with 2,000 tuning and 10,000 sampling steps per chain) were checked for convergence, and for each parameter the effective sample size (ESS) > 10,000 with Gelman-Rubin $\hat{R}\approx 1.000$. We use the 95% high-density interval (HDI) as the credible interval estimator: this provides the boundaries of the smallest interval within the probability distribution that contains 95% of the probability density. For model comparison, we additionally provide the Watanabe–Akaike information criterion (WAIC) score, which measures the out-of-sample prediction accuracy.

#### Hierarchical Bayesian Model With Multiple Explanatory Variables

##### Pass difficulty variable

The context of each pass (e.g., pass length, location) varies across observations. We control for these contextual factors by adding a model variable capturing the difficulty of each pass.

We define *pass difficulty*, *d* ∈ [0, 1], as the conditional probability, *Pr*⁡(*Pass*|*Context*), of completing a pass given various contextual factors (Table 1). *d* near 0 indicates a harder pass; and near 1 indicates an easier pass. To create *d*, we used a random forest (RF) model to fit 12 features to the target variable *y*_*i*_, the pass outcome.

**TABLE 1:** Pass difficulty features.

Feature	Possible values
Pass location (*x*, *y*)	−60.0≤ *x*≤60.0−45.0≤y≤45.0
Transformed *x*-position, *x*′	0≤*x*≤ 60.0
Pass distance, d	d > 0
Pass angle, θ	−π < θ≤π
Transformed pass angle, θ′	0≤θ′≤ 1
Pass type	Ground, aerial
One-touch pass	True, false
Body orientation of passer	Front, sideways, backward
Opposition defensive line in front of passer	Attacking, midfield, defensive
Number of passes, n, in the possession chain until the given pass	n≥0

We used a single variable to encapsulate passing context for two key reasons:

1. **Model simplicity:** our focus is to have an appropriate measure of pass difficulty (i.e., develop a model that learns the conditional probability distribution *Pr*⁡(*Pass*|*Context*)), not to analyze precisely why a pass is difficult;
2. **Computational efficiency:** with a single variable, we have fewer parameter estimates to make for our scanning model, which is important when running the Bayesian hierarchical model, which is computationally intensive.

We chose an RF model for multiple reasons. First, we want to amalgamate contextual factors to create the control variable, *d*, without needing to prescribe relationships between factors—RF models easily provide complexity (linear and non-linear). Second, RF models are quick to cross-validate and tune (i.e., grid-search optimization). Finally, and most importantly, we can calibrate and extract probability outputs from RF models. We needed to create appropriate input features (Table 1) in order to correctly fit to the conditional probability, *Pr*⁡(*Pass*|*Context*), which we describe below.

###### Pass location and body orientation

Positional and body orientation data were hand-tagged by professional coders from StatDNA, LLC (c.f. section *Context*). Pass location (Table 1) is the player’s *x*,*y* position when passing the ball (*x*-direction positive from the defensive-third to the attacking-third; *y*-direction positive from the left-wing to the right-wing). Pitch locations of both the passer and receiver were coded and transformed to a normalized range.

Body orientation of the passer at the time of their pass was coded as follows: forward (if body orientation < ± 45°), sideways (45° ≤ body orientation ≤ 135° or −45° ≥ body orientation ≥ −135°), or backward (| body orientation| > ± 135°); where 0° is the positive x-direction. This orientation was separate to that previously used (see section *Performance With the Ball*), and here used only within the context of the hierarchical Bayesian model (see *Modeling Pass Completion*).

###### Pass distance, angle, transformed angle, and transformed *x*-position

We calculated the pass distance (c.f. see section *Context*) and pass angle using the pitch locations of the passer and receiver. We also derived two additional features. First, we transformed the pass angle to θ′, where

$$\theta^{{}^{\prime }}=\sin\left(\frac{\theta }{2}\right).$$

Here, θ′ as a measure of left-right pass asymmetry (θ′ = 0 when a pass is perfectly from left to right; θ′ = 1 when a pass is perfectly from right to left).

Second, we transformed the *x*-direction to *x*′, where

$$x^{\prime }=60.0-\left|x\right|$$

Here, *x*′ measures the absolute *x* distance to an end-line of the pitch (offensive or defensive).

###### Pass detail

We included features relating to the pass (Table 1): the pass type; a flag indicating whether the pass was a one-touch pass; defensive line faced by passer; and the number of passes by the team in possession until the given pass.

##### Model description

We add the pass difficulty variable (see section *Pass Difficulty Variable*), *d*_*i*_, into our existing Bayesian hierarchical model (c.f. section *Hierarchical Bayesian Model With a Single Explanatory Variable*) with associated parameter γ, as follows:

(3) $$\eta_{i|s}=\alpha_{s}+\beta_{s}\,v_{i|s}+\gamma \,d_{i}$$

Unlike α_*s*_,β_*s*_,we do not condition γ on player *s*, because we assume that pass difficulty (a proxy for passing context) is the same for any passer. That is, a difficult pass is difficult for any player, but better players (with higher α_*s*_) will have a better chance of completing that pass. We assume γhas a Normal prior distribution for the same reasons as for μ_α_ and μ_β_ (c.f. section *Model Description*).

## Results

### General

The players performed on average 3.0 scans (±2.1) in the last 10 s before receiving the ball, giving a mean scan frequency of 0.44 scans/s (±0.30) (note that when the team won the ball or there was a set play within those 10 s, the time interval was shorter than 10 s).

### Contextual Factors’ Influence on Scanning

#### Positional Role and Scan Frequency

**FIGURE 2:**
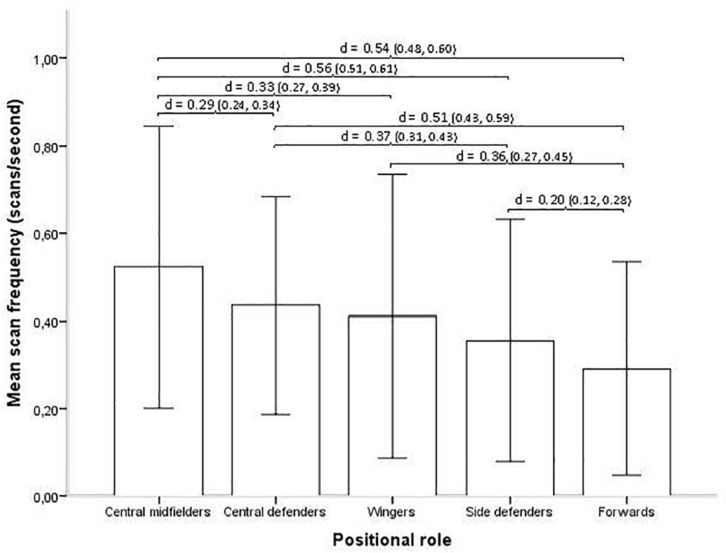
Positional role and mean scan frequency (with SD error bars). Brackets indicate all significant relationships with an effect size >0.20, with effect size *d* and 95% confidence interval (CI) in parentheses.

Scan frequency varies significantly with different positional roles on the team, with central midfielders showing the highest mean frequency and forwards the lowest mean frequency (Kruskal–Wallis *H* = 669.97, *p* < 0.001, see Figure 2). The effect size is *d* = 0.55, which is considered a medium effect (Cohen, 1988). *Post hoc*, pairwise comparison Dunn tests show the scan frequencies for all positional roles were significantly different from each other (all Bonferroni adjusted *p*-values <0.002) with effect sizes ranging from trivial (*d* = 0.16, central defenders and wingers) to medium (*d* = 0.56, central midfielders and side defenders).

#### Opponent Pressure and Scan Frequency

**FIGURE 3:**
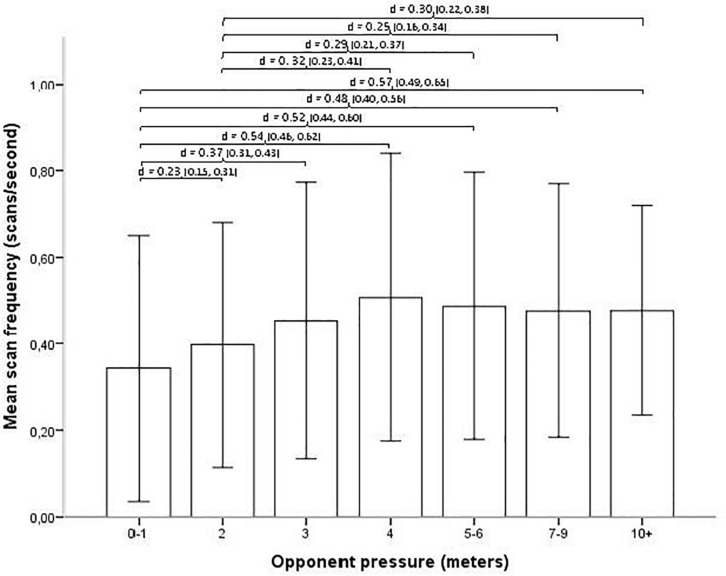
Opponent pressure and mean scan frequency (with SD error bars). Brackets indicate all significant relationships with an effect size >0.20, with effect size *d* and the 95% confidence interval (CI) in parentheses.

The scan frequency appears relatively low in situations where the opponent pressure is high (closest opponent being 0–1 m away when receiving the ball), and then progressively higher when pressure is lower (closest opponent is further away), until the closest opponent is about 4 m away where a further increase in distance is not associated with an increase in scan frequency (see Figure 3). A Kruskal–Wallis test shows that the difference for pressure is significant (*H* = 319.90, *p* < 0.001). The effect size *d* = 0.37 is small. *Post hoc* pairwise comparison Dunn tests show that the scan frequency for the two highest degrees of pressure (0–1 and 2 m) are different from each of the other degrees of pressure (*p* < 0.002), the third highest pressure (3 m) is different from each of the other degrees of pressure (*p* < 0.003) except 7–9 m, and the four lower degrees of pressure (4, 5–6, 7–9, and 10+ m) are only different from each of the three highest degrees of pressure (0–1, 2, and 3 m) (*p* < 0.003) (all *p*-values Bonferroni adjusted for multiple tests). The effect sizes range from trivial (*d* = 0.04 for 5–6 m compared to 7–8 m) to medium (*d* = 0.57, for 0–1 m compared to 10+ m).

#### Pitch Location and Scan Frequency

**FIGURE 4:**
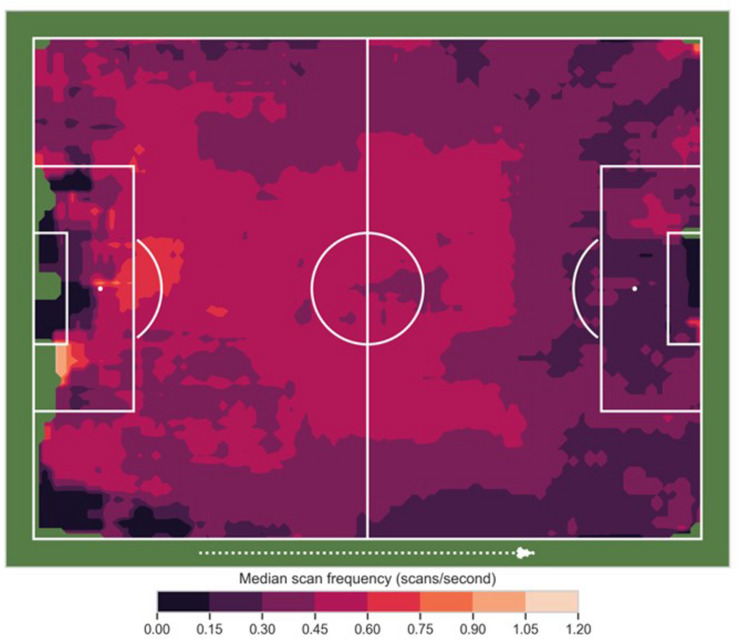
Pitch location and scan frequency. The attacking direction is normalized from left to right (dotted arrow). Colors at a given location (*x,y*,) show the median search frequency calculated from a 12 × 8-m box centered on that point.

For passing events, on average, players scan above the 75th percentile (0.6 scans/s) when passing around their own 18-yard box, in the central area between their penalty spot and the top of the “D” (Figure 4). Scanning tends to be above average (>0.45 scans/s) but below the 75th percentile consistently through the left channel (we define the channel to be the width between 6-yard box and 18-yard box, here traversing the pitch from defense to attack). The right channel does not show an exact symmetry of the left channel with scanning dropping off in both the defensive third and attacking third.

Scanning decreases below average near the boundary areas (<0.3 scans/s), especially in the attacking third. It drops almost toward 0 scans/s near the defensive and attacking 6-yard box and near the right defensive corner flag. There is also a pronounced drop-off in scanning from the midfield third to the attacking third.

#### Game State and Scan Frequency

Game state, for the purpose of this study, was represented by game standing and accumulated game time. Game standing (whether the team at that moment is winning, losing, or drawing) was significantly, but marginally linked to scan frequency (Kruskal–Wallis *H* = 7.50, *p* = 0.024). *Post hoc* pairwise comparison Dunn tests show that scan frequency was higher when the team is losing (*M* = 0.46 scans/s ± 0.29, *N* = 912) than when the team is drawing (*M* = 0.44 scans/s ± 0.30, *N* = 4,102) (adjusted *p* = 0.020), but the effect size *d* = 0.08 suggests that this is a trivial effect. There were no significant differences with when the team is winning (*M* = 0.45 scans/s ± 0.31, *N* = 4,296) (both adjusted *p*-values >0.013) (effect sizes *d* < 0.06).

**FIGURE 5:**
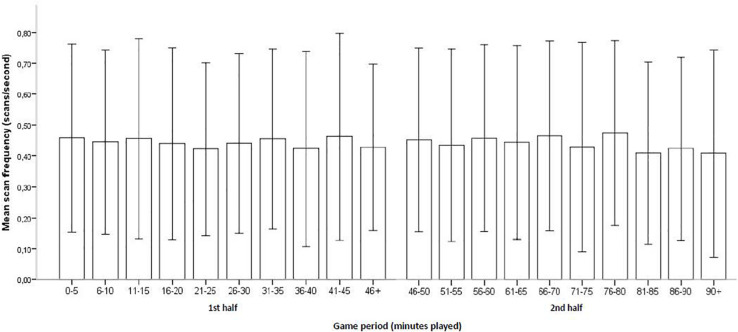
Accumulated game time and mean scan frequency (with SD error bars).

For game time, scanning frequency was relatively stable throughout the different time phases in the first half of the games (*H* = 8.99, *p* = 0.439), but less stable in the second half with a significant difference between the time phases (*H* = 24.06, *p* = 0.004) (*N* = 8,733 possessions, where only the players who started the game were included in the analysis, see Figure 5). However, the effect size, *d* = 0.12, is trivial. *Post hoc* pairwise comparison Dunn tests, where we use Bonferroni adjustments to control for the large number of tests, showed no significant differences. However, there was a trend for a difference between 76 and 80 min and 81–85 min (adjusted *p* = 0.062, effect size *d* = 0.21), and between 76 and 80 min and 90+ min (adjusted *p* = 0.063, effect size *d* = 0.23).

When we combined game standing and game time, we observed a similar pattern when the team is winning, with no differences for the first half (*H* = 13.29, *p* = 0.15, *N* = 1,511 possessions), but a difference for the second half (*H* = 23.85, *p* = 0.005, *N* = 2,645 possessions, where the *post hoc* pairwise comparisons show no significant differences). The effect size was trivial, *d* = 0.15. For possessions where the team is drawing, there were no differences in the first half (*H* = 11.30, *p* = 0.256, *N* = 2,669 possessions) or in the second half (*H* = 13.18, *p* = 0.155, *N* = 996 possessions). However, for possessions when the team is losing, there was no difference for the second half (*H* = 4.98, *p* = 0.836, *N* = 460 possessions), but there was a difference for the first half where the scan frequencies tended to drop toward the end of the half (*H* = 25.69, *p* = 0.001, *N* = 452 possessions, effect size *d* = 0.47). The *post hoc* pairwise comparisons for the first half showed significant differences between 45+ min and 5–10 min (adjusted *p* = 0.005, effect size *d* = 1.25), and between 45+ min and 31–35 min (adjusted *p* = 0.048, effect size *d* = 0.84). Both these effect sizes are considered large (Cohen, 1988), but the sample sizes are very small (e.g., only 35 possessions for the 45+-min condition) and the result needs to be interpreted with much caution.

### Scan Frequency and Performance

#### Scan Frequency and Action Direction

Analysis of the players’ scanning frequency prior to their last action in a ball possession showed that players scanned more frequently prior to actions directed forward (*M* = 0.46 scans/s ± 0.31, *N* = 5,776), compared to sideward (0.43 scans/s ± 0.31, *N* = 663) and backward (0.42 scans/s ± 0.30, *N* = 2,860) (Kruskal–Wallis *H* = 30.602, *p* < 0.001, effect size *d* = 0.11). Pairwise comparison with Bonferroni corrected adjusted significance values showed a difference only between passes directed forward and backward (*p* < 0.001). The effects sizes were between *d* = 0.06 and *d* = 0.12, which suggests these were trivial effects.

#### Scan Frequency and Action Type

Players had the highest scanning frequency when their last action was a pass (*M* = 0.45 scans/s ± 0.30, *N* = 8,760), compared to a dribble (*M* = 0.39 scans/s ± 0.30, *N* = 289), receiving the ball (*M* = 0.35 scans/s ± 0.31, *N* = 160), and finishing (*M* = 0.27 scans/s ± 0.24, *N* = 207) (Kruskal–Wallis *H* = 114.98, *p* < 0.001, effect size *d* = 0.22). Pairwise comparison with Bonferroni corrected adjusted p-values show significant differences between passing and finishing (*p* < 0.001, effect size *d* = 0.19), passing and receiving (*p* < 0.001, effect size *d* = 0.10), passing and dribbling (*p* < 0.001, effect size *d* = 0.08), and between dribbling and finishing (*p* < 0.001, effect size *d* = 0.42).

**FIGURE 6:**
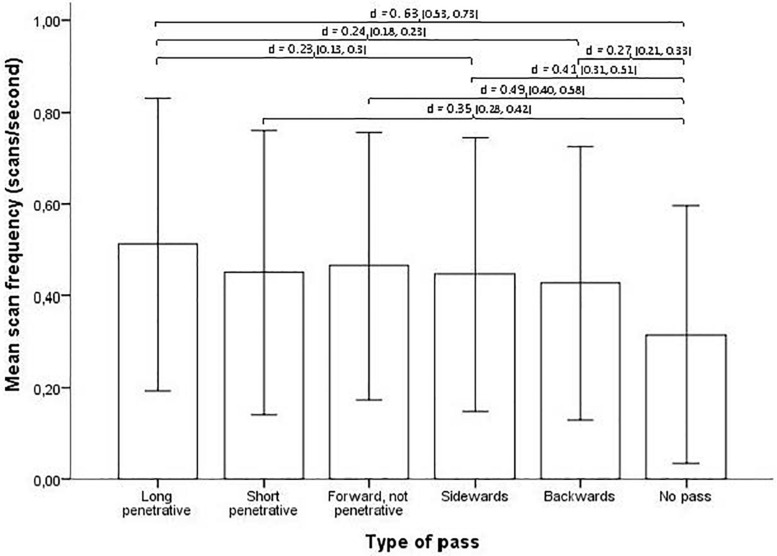
Type of pass and Mean scan frequency (with SD error bars). Brackets indicate all significant relationships with an effect size >0.20, with effect size *d* and the 95% confidence interval (CI) in parentheses.

Breaking down the last actions in a possession into different passing types, players scanned most frequently prior to long penetrative passes and less with passes that were shorter and/or less directed forward (see Figure 6) (Kruskal–Wallis *H* = 64.751, *p* < 0.001). Pairwise comparison with Bonferroni corrected adjusted significance values show significant differences between long penetrative passes and backward passes (*p* < 0.001, effect size *d* = 0.24), long penetrative passes and sideward passes (*p* < 0.001, effect size *d* = 0.23), long penetrative passes and short penetrative passes (*p* < 0.001, effect size *d* = 0.19), as well as between “forward, not penetrative passes” and backward passes (*p* < 0.001, effect size *d* = 0.15). In addition, the pairwise comparisons show significant differences between possessions where no pass is given and all the other instances of passes (all adjusted *p* < 0.001), with the effect sizes *d* ranging between 0.27 and 0.63.

#### Scan Frequency and Successful Actions

Players scanned significantly higher when possession was maintained after their actions with the ball (*M* = 0.46 scans/s ± 0.30) than when possession was lost after their action (*M* = 0.37 ± 0.30) (Mann-Whitney *U* = 4,540,860, *p* < 0.001, *N* = 9,510 possessions, effect size *d* = 0.20). For those possessions where the players end up playing a pass (*N* = 8,825 possessions), they also scanned higher when their passes reached a teammate (i.e., pass completed, *M* = 0.46 scans/s ± 0.30) than when their passes did not reach a teammate (i.e., pass not completed, *M* = 0.40 scans/s ± 0.30) (Mann-Whitney *U* = 3,649,383, *p* < 0.001, effect size *d* = 0.15).

### Modeling Pass Completion

#### Hierarchical Bayesian Model With a Single Explanatory Variable

Our hierarchical model generates 27 α_*s*_ and 27 β_*s*_ pairs; one per player (see section *Model Description*). These specific player parameters are assumed to be distributed from a prior normal distribution, described by μ_α_,σ_α_, and μ_β_,σ_β_, which we report here (we do not report the specific player parameter estimates, as they are not of interest compared to the estimates of the group parameters). Estimates for the group-level intercept term are: μ_α_ = 2.07 ± 0.11[1.86, 2.29] and σ_α_ = 0.49 ± 0.10[0.30, 0.67]. Estimates for the group-level scanning coefficient are: μ_β_ = 0.16 ± 0.06[0.03, 0.28] and σ_β_ = 0.20 ± 0.07[0.06, 0.34]. The σ_β_ ESS = 3,889.67, indicating slightly less robustness in the HDI estimate (for all other parameters the ESS ≥ 10,000). The model WAIC = 5,307.21.

#### Hierarchical Bayesian Model With Multiple Explanatory Variables

We added pass difficulty, *d*, to the hierarchical model [see *Model Description* (under section *Hierarchical Bayesian Model With Multiple Explanatory Variables*)]. Estimates for the group-level intercept term are: μ_α_ = 2.44 ± 0.11[2.22, 2.67] and σ_α_ = 0.47 ± 0.09[0.31, 0.67](ESS = 1,908.7 and 4,386.2 for μ_α_,σ_α_ respectively). Estimates for the group-level scanning coefficient are: μ_β_ = 0.13 ± 0.06[0.02, 0.24] and σ_β_ = 0.12 ± 0.07[0.01, 0.24] (ESS = 3,368.1 and 1,058.6 for μ_β_,σ_β_ respectively). Estimates for the pass difficulty coefficient γ = 0.97 ± 0.03[0.91, 1.03] (ESS = 5, 551.2). For all other parameters, the ESS was ≥3,000 with 40% of parameters ≥10,0000.0. The model WAIC = 4,122.25, which is lower compared to our hierarchical model with a single variable (WAIC = 5,307.21; see section *Hierarchical Bayesian Model With a Single Explanatory Variable*), thus indicating better pointwise out-of-sample predictive accuracy.

**FIGURE 7:**
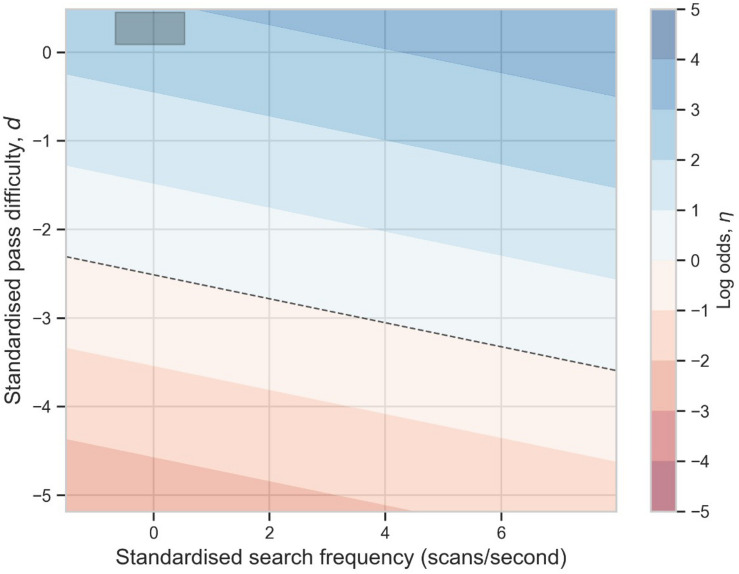
Contour plot of the log-odds, η, plotted for different standardized values of the search frequency, *v*, and pass difficulty, *d*, using the coefficients μ_α_, μ_β_ and γ (Eq. 3). The 25th and 75th percentile values for *v* and *d* are shown (dark gray shaded box). Probability of pass completion is ≥50% above and < 50% below the black dashed line.

The log-odds of completing a pass, η, decreases as passes become more difficult (*d*→0; Figure 7). The contours have a negative slope (c.f. black dashed line): when pass difficulty is kept constant, the more a player scans, the greater the probability of completing a pass.

However the 25–75% percentile domain, within which most scan frequencies and pass difficulties lie (dark shaded area), is small relative to the practical range of η (η≈2.2→*p*≈0.9; η≈−2.2→*p*≈0.1; c.f. section *Motivation*). The magnitude of the group intercept term, μ_α_, dominates the latent variable equation (Eq. 3) and its variability is on the same scale as the group scanning coefficient, μ_β_. Pass difficulty, *d*, plays the second largest role in pass completion. This indicates that the advantage of increased scanning is much smaller than players’ intrinsic technical abilities and the passing context.

## Discussion

The study was conducted to learn more about how 27 EPL professional football players use scanning prior to their individual ball possessions during 21 competitive games, across a season. We hypothesized that scanning frequencies increase in certain positions and situations, specifically when playing centrally in the field and under loose pressure from opponents, and that scanning is related to performance with the ball. Overall, the results supported these hypotheses, and showed that these players’ scanning varied with different types of contextual demands (i.e., positional role, opponent pressure, pitch location, and to some extent game state), although some of the differences were small (i.e., effect sizes between 0.2 and 0.5). Moreover, scanning prior to receiving the ball was linked to performance with the ball, in that players in situations where they scanned more produced more passes, more long penetrative passes and more successful actions with the ball, but these effects were also quite small. Our more statistically sophisticated pass completion models show that scan frequency played a small, positive role for players completing their passes. Here, we will discuss these findings more in detail.

### Contextual Influences

Players that hold different positional roles showed different degrees of scanning frequency. Defenders and midfielders with central positions (central midfielders and central defenders) displayed higher scanning frequency than players along the sides of the field (particularly side defenders, but also wingers, even though this difference was smaller) or players relatively higher up in the field (forwards). This is consistent with the finding from a previous study that central players scanned more than wide players when they, or their team, had possession of the ball (McGuckian et al., 2020), and with another study showing that central midfielders and central defenders are the most prominent playing positions when building an attack in football (Clemente et al., 2015).

Although we here will conjecture that players in certain positions scan less than others because of logical requirements from the game, it is possible that they scan less in certain situations even though they should scan more. With that said, an explanation for this finding could be that centrally (as compared to more peripherally) located players are constantly surrounded by both teammates and opponents, which logically necessitates more frequent scanning to obtain and update the informational basis for one’s actions. Previous research has shown that players’ space exploration ability is influenced by space restrictions (Gonçalves et al., 2017). Hence, the inherent space constraints for peripherally positioned players are likely also to influence their scanning ability. Moreover, players located along the edges of the field can logically restrict the orientation of their scanning to one direction, inward in the field (i.e., they do not have to scan for information in the direction of the sideline, as there is no relevant information outside the field).

Forwards scan with a lower frequency than the other positional roles, and we hypothesize a few possible explanations for this. First, forwards are likely to receive the ball in tighter areas that are more guarded by opponents. If ball receiving precision drops here, and it would seem likely to do so if the forward takes his eyes off the ball at an inopportune time, the ball is likely to be lost. Second, forwards may scan less in the seconds before they receive the ball because they typically are so close to defenders that they perceive where they are without having to scan (e.g., from physical contact or from peripheral vision) and/or because a prearranged game plan/game model stipulates some of the likely surroundings making scanning less necessary. Third, forwards contribute less than any other position in the attacking build up (Clemente et al., 2015). Consequently, when forwards are about to receive the ball, their visual attention is likely more narrowly directed toward finishing an attack (with less scanning for surrounding passing options) compared to central midfielders who will scan for teammates in order to build up an attack (Clemente et al., 2015).

The results for location in the field and scanning are to a large extent aligned with the results for positional role and scanning. As indicated in Figure 4, scanning frequency is relatively low in both far ends of the field. There is a distinct drop from the midfield third to the attacking third, and there are somewhat lower scan frequencies along the sidelines as compared to a central channel between the two goals. Scanning is relatively high in several sections of the players’ own half, possibly because players on the team in possession of the ball have to be very aware of their opponents, given that losing the ball here might have disastrous consequences. At the same time, they typically have the game in front of them, more space around them and time to scan and fully prepare the reception of a potential pass. Interestingly, only parts of these results are in line with a recent study where elite youth players indeed scanned more frequently in central as opposed to wide areas of the field, but less frequently in the middle third than in the back and front third of the field (McGuckian et al., 2020). It is possible that the difference in performance level (professional Premier League players vs elite youth players) may account for this difference.

The results for opponent pressure and visual scanning support the results for positional role and field location, although with the biggest difference between situations under tight pressure (receiving the ball with closest opponent being 0–1 m away) and situations under considerably looser pressure (i.e., 4 m or more away) (medium effect sizes). That the players in this study scan less frequently when the closest opponents are less than 1 m away could be due to the heightened risk of taking their eyes off the ball when opponents are near. Also, when defenders are that close, the players receiving the ball may already be aware of the defensive threat (due to physical body contact or peripheral awareness of the defender), thus reducing the need to scan.

The players in our study seem to scan significantly, but marginally less frequently toward the end of the second half of a game, as compared to earlier in the second half. This would be consistent with studies showing that football players’ running seems to drop toward the end of the game (Carling et al., 2015). The same drop in scanning frequency was evident under conditions where one’s team was in a lead. When the team was behind in the score, there was no drop in the second half, but indeed a drop toward the end of first half. Certainly, our data on this topic is far from conclusive and our interpretations are extremely tentative. This would be enhanced by structured input from coaches, and more focused research is needed to be able to say more about some of the mechanisms that may underlie these observations.

### Scanning and Performance

The main objective with our study was to examine the potential role that scanning plays for different types of performance with the ball. In general, scanning frequency was associated with more passes (compared to dribbles and shots), more long and forward passes, and more dribbles (compared to shots). Even though most of these effects were small, the results might imply that engaging in scanning lead players to more effectively detect and utilize progressive/forward-passing opportunities. Such association between scanning and type of action would be consistent with those from previous studies on elite youth players (Eldridge et al., 2013) and semi-elite adult players (McGuckian et al., 2018) showing that scanning is associated with more forward passes.

Importantly, with our Bayesian hierarchical model, the data we have collected adds evidence toward the hypothesis that increased scanning increases the probability of completing passes. This conclusion is maintained when controlling for differences between players and the difficulty of passes. Lowest case estimates of μ_β_ suggest that for ≈53% of players scanning plays a positive role in pass completion (*Z* = 0.08). Highest case estimates suggest that for ≈100% of players scanning plays a positive role (*Z* = 24.0). Mean estimates suggest that for ≈86% of players scanning plays a positive role (*Z* = 1.08). Thus, the more players scan prior to receiving the ball, the more likely they are to play a successful pass to a teammate. This agrees with results from previous studies at the same level of performance (Jordet et al., 2013) and could be consistent with the finding that higher scanning frequency is associated with faster response time (McGuckian et al., 2019), which is likely a sign that increased rate of scanning produces more accurate perception which would positively affect pass completion. However, the result is counter to results from field studies with players at a lower level of performance that do not find this relationship (Eldridge et al., 2013; McGuckian et al., 2018).

Based on the theoretical premise that active perception is better than passive perception (Adolph et al., 2000), it makes sense that more extensive visual exploration of one’s surroundings is linked to more accurate perception and subsequent performance toward the same surroundings. Ecological psychologists will argue that a major advantage of engaging in exploratory scanning activity is that the payoff in terms of information located and used can be quite large, yet the energetic expenses are minimal (Reed, 1996). More specifically, this is in line with Gibson’s (1979) concept of affordances which states that action possibilities can be found through actively exploring the environment, and it is only when a player continually updates himself that he is able to see which opportunities are opening up and closing down (Marsh and Meagher, 2016). Interestingly, extensive research has shown that individuals are not only attuned to their own affordances but also sensitive to the action possibilities of other individuals in their environment (i.e., teammates and opponents) (Marsh and Meagher, 2016). Hence, by scanning more, players will be attuned to more opportunities for action for themselves as well as having an increased awareness of the affordances of their direct opponent and teammates, which in turn should lead to an enhanced prospective control of their actions (Fajen et al., 2008).

However, our predictive models suggest that while increased scanning conferred a small advantage on pass completion, this was small. A player’s technical ability and the difficulty of a pass (embedded in a team’s familiar game model) are likely still primarily responsible for pass completion. Researchers are advised to continue to examine the extent to which scanning may be related to performance, and the different mechanisms that may support such a relationship. This includes pursuing research on aspects around scanning that we were not able to focus on here, such as scan excursion (which would say something about the scope of information gathered in each scan McGuckian et al., 2018, 2019) and defensive scanning (scanning when the other team has the ball).

### Limitations

There are several limitations with this study that suggest the results need to be interpreted with caution.

First, even though the number of individual ball possessions analyzed is relatively high (almost 10,000), the players in the study all came from only one team, and the results are not necessarily representative for other players and teams, even at the same high level of performance. The particular team that was analyzed in this study is known to play possession-based football, and it is possible that an analysis of players on teams that follow a different game model would give different results.

Second, all the games were played at home, and given that we know the home advantage has a robust impact on results in professional football games (including those in the EPL, Pollard and Gómez, 2014), it is possible that these players would have behaved somewhat differently when they play away.

Third, although a very strict observation protocol was followed and interreliability test scores were very good, manually coding this type of behavior in a fluid and complex field event will undoubtedly be associated with measurement errors. In our position assessments, we did not fully account for the instances where a player changed position late in the match, which should be better captured in future research. Also, future researchers need to continue to improve and refine the quality of scanning measurements, which includes learning more about the conditions where scanning is easy and difficult to accurately assess. Related to that, there is a need to explore the cutoff values for the time interval in which scanning is measured, as other intervals than 10 s could be more adaptive in certain game phases and situations.

### Practical Applications

Despite methodological limitations, we can suggest some general applied implications from this study. The results provide some support that scanning is a process that practitioners could focus on to help football players improving their pickup of visual information, to facilitate performance. Previous studies have shown that even relatively short interventions have the capacity to help football players at the professional (Jordet, 2005b) and elite youth academy levels (Pocock et al., 2017) increase their rate of visual scanning and that this again might positively support performance (i.e., improvements in performance were noted for some of the players in both those studies). Indeed, coaches have started to integrate exercises on scanning into their practices (e.g., Jozak and Kepcija, 2017; Pulling et al., 2018) and emerging technological innovations are addressing this skill (e.g., the Footbonaut, Beavan et al., 2018). Our study lends some tentative support to continue work in this direction.

In general, as sport psychology practitioners we seek to support athletes’ ability to place, change and control their attention. Having insights into how they go about gaining information is a fruitful pathway into performance enhancement discussions with players and coaches. Some of the practical questions to players could be, what do coaches want them to look for? What cues? When do coaches want them to look, and when not to? What are the crucial moments within a game that interest coaches and to what extent do players gain or miss crucial information in split-second moments that often define a game? Similarly, practitioners can facilitate the integration of the behaviors into exercises and game-based activities in training. Further, coaches and analysts often analyze football games, in the moment and after games, using video technology. While in the future we may have the athletes’ own eye view, on ground level, at this time, analysis is often done from a bird’s-eye view. Inferring from above (often in comfort on a screen), what goes on for a player is very different looking down than on the ground in the moment and might arguably lead to unrealistic and unfair interventions that do not represent the actual experience of the player. With this study, we do not wish to feed this divide but instead find innovative ways to close it.

## Conclusion

Elite professional football players competing in an EPL team engaged in frequent visual scanning behaviors in the seconds prior to receiving the ball. There were some positional and contextual differences in scanning, which can be explained by the requirements of different phases and aspects of the game. Through a statistically sophisticated model, our data added evidence toward a positive, albeit small, relationship between scanning and pass completion, suggesting that scanning can play a positive role for pass completion. With that said, particularly given that many of the differences we uncovered were relatively small or modest, we do not believe or claim that scanning is the conclusive variable associated with football performance. Innumerable and immeasurable factors can affect a player and team performance at any given time (on or off the grass). Instead our interest with this article lies in exploring this one variable, on the grass, in the game, knowing its incompleteness, but also its future potential to be linked with other multidisciplinary data that could lead to fascinating and insightful dialogue and interventions with players and coaches if the marriage of technology and human relationships continue to strengthen.

## Data Availability Statement

The data generated for this study are available upon request to the first author.

## Ethics Statement

The studies involving human participants were reviewed and approved by the Norwegian Centre for Research Data (NSD, project number 57718). Written informed consent for participation was not required for this study in accordance with the national legislation and the institutional requirements.

## Author Contributions

GJ contributed to conceptualization, administration of data collection, data analysis and writing the manuscript. KMA contributed to conceptualization, data collection, parts of the data analysis and writing the manuscript. DNP contributed to conceptualization, data collection, data management, and administration of data analysis. AW contributed to data management and writing the manuscript. AT contributed to the data analysis, modeling and writing the manuscript. AM contributed to data analysis and writing the manuscript. AI contributed to data analysis and writing the manuscript. DP contributed to conceptualization, data analysis, and writing the manuscript. All authors contributed to the article and approved the submitted version.

## Conflict of Interest

GJ, AT, AM, AI, and DP were affiliated with or employed by Arsenal FC. The remaining authors declare that the research was conducted in the absence of any commercial or financial relationships that could be construed as a potential conflict of interest. The authors declare that this study received part of the funding from Arsenal FC. Authors affiliated with or employed by the funder (GJ, AT, AM, AI, and DP) were involved in the study design, analysis, interpretation of data, and the writing of this article. It was agreed at the outset that the specific findings i.e. positive or negative, would not impact the decision to submit it for publication.

## References

[B1] Adolph K. E. Eppler M. A. Marin L. Weise I. B. Wechsler Clearfield M. (2000). Exploration in the service of prospective control. *Infant. Behav. Dev.* 23 441–460. 10.1016/S0163-6383(01)00052-2

[B2] Araújo D. Davids K. Passos P. (2007). Ecological validity, representative design, and correspondence between experimental task constraints and behavioral setting: comment on rogers, Kadar, and Costall (2005). *Ecol. Psychol.* 19 69–78. 10.1080/10407410709336951

[B3] Beavan A. Fransen J. Spielmann J. Mayer J. Skorski S. Meyer T. (2018). The Footbonaut as a new football-specific skills test: reproducibility and age-related differences in highly trained youth players. *Sci. Med. Football* 3 177–182. 10.1080/24733938.2018.1548772

[B4] Cañal-Bruland R. Lotz S. Hagemann N. Schorer J. Strauss B. (2011). Visual span and change detection in soccer: an expertise study. *J. Cogn. Psychol.* 23 302–310. 10.1080/20445911.2011.496723

[B5] Carling C. Gregson W. McCall A. Moreira A. Wong D. Bradley P. (2015). Match running performance during fixture congestion in elite soccer: research issues and future directions. *Sports Med.* 45 605–613. 10.1007/s40279-015-0313-z 25694027

[B6] Cicchetti D. V. (1994). Guidelines, criteria, and rules of thumb for evaluating normed and standardized assessment instruments in psychology. *Psychol. Assess.* 6 284–290. 10.1037/1040-3590.6.4.284

[B7] Clemente F. Martins F. Del Wong P. Kalamaras D. Mendes R. (2015). Midfielder as the prominent participant in the building attack: a network analysis of national teams in FIFA World Cup 2014. *Int. J. Perf. Anal. Spor.* 15 704–722. 10.1080/24748668.2015.11868825

[B8] Cohen J. (1960). A coefficient of agreement for nominal scales. *Educ. Psychol. Meas*. 20 37–46. 10.1177/001316446002000104

[B9] Cohen J. (1988). *Statistical Power Analysis for the Behavioral Sciences.* New York, NY: Routledge Academic.

[B10] Eldridge D. Pulling C. Robins M. T. (2013). Visual exploratory activity and resultantbehavioural analysis of youth midfield soccer players. *J. Hum. Sport Exerc.* 8 560–577. 10.4100/jhse.2013.8.Proc3.02

[B11] Fajen B. A. Riley M. A. Turvey M. T. (2008). Information, affordances, and the control of action in sport. *Int. J. Sport Psychol.* 40 79–107.

[B12] Gelman A. (2006). Prior distributions for variance parameters in hierarchical models (comment on article by Browne and Draper). *Bayesian Anal.* 1 515–534. 10.1214/06-ba117a

[B13] Gibson J. J. (1966). *The Senses Considered as Perceptual Systems.* Boston: Houghton Mifflin.

[B14] Gibson J. J. (1979). *The Ecological Approach to Visual Perception.* Boston: Hougthon Mifflin.

[B15] Gonçalves B. Esteves F. H. Ric A. Torrents C. Sampaio J. (2017). Effects of pitch area-restrictions on tactical behavior, physical, and physiological performances in soccer large-sided games. *J. Strength Cond. Res.* 31:1700. 10.1519/JSC.0000000000001700 27806007

[B16] Hallgren K. A. (2012). Computing inter-rater reliability for observation data: an overview and tutorial. *Tutor. Quant. Methods Psychol.* 8 23–34. 10.20982/tqmp.08.1.p023 22833776 PMC3402032

[B17] Helsen W. F. Starkes J. L. (1999). A multidimensional approach to skilled perception and performance in sport. *Appl. Cogn. Psychol.* 13 1–27. 10.1002/(sici)1099-0720(199902)13:1<1::aid-acp540>3.0.co;2-t

[B18] Hrycaiko D. Martin G. L. (1996). Applied research studies with single-subject designs: why so few? *J. Appl. Sport Psychol.* 8 183–199. 10.1080/10413209608406476

[B19] Jordet G. (2005a). “Applied cognitive sport psychology in team ball sports: an ecological approach,” in *New Approaches to Sport and Exercise Psychology*, eds Stelter R. Roessler K. K. (Aachen: Meyer & Meyer Sport), 147–174.

[B20] Jordet G. (2005b). Perceptual training in soccer: an imagery intervention study with elite players. *J. Appl. Sport Psychol.* 17 140–156. 10.1080/10413200590932452

[B21] Jordet G. Bloomfield J. Heijmerikx J. (2013). The hidden foundation of field vision in English Premier League (EPL) soccer players, *Paper Presented at the MIT Sloan Sports Analytics Conference*, Boston.

[B22] Jozak R. Kepcija I. (2017). *Croatian Football Federation - Development Curriculum.* Zagreb: Vivid & Shine j.d.o.o.

[B23] Landis J. R. Koch G. G. (1977). The measurement of observer agreement forcategorical data. *Biometrics* 33 159–174. 10.2307/2529310 843571

[B24] Linke D. Link D. Lames M. (2020). Football-specific validity of TRACAB’s optical video tracking systems. *PLoS One* 15:e0230179. 10.1371/journal.pone.0230179 32155220 PMC7064167

[B25] Mann D. L. Causer J. Nakamoto H. Runswick O. R. (2019). “Visual search behaviours in expert perceptual judgements,” in *Anticipation and Decision Making in Sport*, eds Williams A. M. Jackson R. C. (London: Taylor & Francis), 59–78. 10.4324/9781315146270-4

[B26] Marsh K. L. Meagher B. R. (2016). “Affordances and interpersonal coordination,” in *Interpersonal Coordination and Performance in Social Systems*, eds Passos P. Davids K. Chow J. Y. (London: Routledge), 245–258.

[B27] McGuckian T. B. Cole M. H. Chalkley D. Jordet G. Pepping G.-J. (2019). Visual exploration when surrounded by affordances: frequency of head movements is predictive of response speed. *Ecol. Psychol*. 31 1–19. 10.1080/10407413.2018.1495548

[B28] McGuckian T. B. Cole M. H. Jordet G. Chalkley D. Pepping G.-J. (2018). Don’t turn blind! the relationship between exploration before ball possession and on-ball performance in association football. *Front. Psychol.* 9:2520. 10.3389/fpsyg.2018.02520 30618946 PMC6295565

[B29] McGuckian T. B. Cole M. H. Jordet G. Chalkley D. Pepping G.-J. (2020). Constraints on visual exploration of youth football players during 11v11 match-play: the influence of playing role, pitch position and phase of play. *J. Sports Sci.* 38 658–668. 10.1080/02640414.2020.1723375 32009533

[B30] Oppici L. Panchuk D. Serpiello F. R. Farrow D. (2017). Long-term practice with domain-specific task constraints influences perceptual skills. *Front. Psychol.* 8:1387. 10.3389/fpsyg.2017.01387 28855883 PMC5557782

[B31] Pinder R. Davids K. Renshaw I. Araújo D. (2011). Representative learning design and functionality of research and practice in sport. *J. Sport Exerc. Psychol.* 33 146–155. 10.1123/jsep.33.1.146 21451175

[B32] Pocock C. Dicks M. Thelwell R. C. Chapman M. Barker J. B. (2017). Using an imagery intervention to train visual exploratory activity in elite academy football players. *J. Appl. Sport Psychol.* 31 218–234. 10.1080/10413200.2017.1395929

[B33] Pollard R. Gómez M. A. (2014). Components of home advantage in 157 national soccerleagues worldwide. *Int. J. Sport Exerc. Psychol.* 12 218–233. 10.1080/1612197X.2014.888245

[B34] Pulling C. Kearney P. Eldridge D. Dicks M. (2018). Football coaches’ perceptions of the introduction, delivery and evaluation of visual exploratory activity. *Psychol. Sport Exerc.* 39 81–89. 10.1016/j.psychsport.2018.08.001

[B35] Reed E. S. (1996). *Encountering the World: Towards an Ecological Psychology.* New York, NY: Oxford University Press.

[B36] Roca A. Ford P. R. McRobert A. P. Williams A. M. (2011). Identifying the processes underpinning anticipation and decision-making in a dynamic time-constrained task. *Cogn. Process.* 12 301–310. 10.1007/s10339-011-0392-1 21305386

[B37] Roca A. Ford P. R. Memmert D. (2018). Creative decision making and visual search behavior in skilled soccer players. *PLoS One* 13:e0199381. 10.1371/journal.pone.0199381 29990320 PMC6039007

[B38] Salvatier J. Wiecki T. V. Fonnesbeck C. (2016). Probabilistic programming in Python using PyMC3. *PeerJ Comput. Sci.* 2:e55 10.7717/peerj-cs.55 PMC10495961 37705656

[B39] Scheibe J. (2019). *Die Optionen im Blick Behalten: die Qualität Einer Aktion Hängtmaßgeblich von der Vororientierung ab.* Available online at: https://www.dfb-akademie.de/vororientierung-die-optionen-im-blick-behalten/-/id-11008663 (accessed March 2, 2020)

[B40] Vaeyens R. Lenoir M. Williams A. M. Mazyn L. Philippaerts R. M. (2007a). The effects of task constraints on visual search behavior and decision-making skill in youth soccer players. *J. Sport Exerc. Psychol.* 29 147–169. 10.1123/jsep.29.2.147 17568064

[B41] Vaeyens R. Lenoir M. Williams A. M. Philippaerts R. M. (2007b). Mechanisms underpinning successful decision making in skilled youth soccer players: an analysis of visual search behaviors. *J. Mot. Behav.* 39 395–408. 10.3200/JMBR.39.5.395-408 17827116

[B42] Williams A. M. Jackson R. C. (2019). Anticipation in sport: fifty years on, what have we learned and what research still needs to be undertaken? *Psychol. Sport Exerc.* 42 16–24. 10.1016/j.psychsport.2018.11.014

